# Weight Gain With Advancing Age Is Controlled by the Muscarinic Acetylcholine Receptor M4 in Male Mice

**DOI:** 10.1210/endocr/bqaf064

**Published:** 2025-04-03

**Authors:** Toshio Takahashi, Yuta Takase, Akira Shiraishi, Shin Matsubara, Takehiro Watanabe, Shinji Kirimoto, Tohru Yamagaki, Masatake Osawa

**Affiliations:** Suntory Foundation for Life Sciences, Bioorganic Research Institute, Kyoto 619-0284, Japan; Suntory Foundation for Life Sciences, Bioorganic Research Institute, Kyoto 619-0284, Japan; Suntory Foundation for Life Sciences, Bioorganic Research Institute, Kyoto 619-0284, Japan; Suntory Foundation for Life Sciences, Bioorganic Research Institute, Kyoto 619-0284, Japan; Suntory Foundation for Life Sciences, Bioorganic Research Institute, Kyoto 619-0284, Japan; Animal Science Business Unit, KAC Co., Ltd., Kyoto 604-8423, Japan; Suntory Foundation for Life Sciences, Bioorganic Research Institute, Kyoto 619-0284, Japan; Department of Regenerative Medicine and Applied Biomedical Sciences, Graduate School of Medicine, Gifu University, Gifu 501-1194, Japan; Center for Highly Advanced Integration of Nano and Life Sciences, Gifu University, Gifu 501-1194, Japan

**Keywords:** acetylcholine, muscarinic receptor, body weight, white adipose tissue, brown adipose tissue

## Abstract

Obesity is characterized by the excessive accumulation of adipose tissue, and it is a serious global health issue. Understanding the pathology of obesity is crucial for developing effective interventions. In this study, we investigated the role of muscarinic acetylcholine receptor M4 (mAChR-M4) in the regulation of obesity in *Chrm4*-knockout (M4-KO) mice. Male M4-KO mice showed higher weight gain and accumulation of white adipose tissue (WAT) with advancing age than the wild-type mice. The M4-KO mice also showed increased leptin expression at both the transcription and the translation levels. RNA sequencing and quantitative reverse transcription polymerase chain reaction analyses of subcutaneous adipose tissues revealed that the expression of WAT marker genes was significantly enhanced in the M4-KO mice. In contrast, the expression levels of brown adipose tissue/beige adipose tissue markers were strongly decreased in the M4-KO mice. To identify the *Chrm4*-expressing cell types, we generated *Chrm4-mScarlet* reporter mice and examined the localization of the mScarlet fluorescent signals in subcutaneous tissues. Fluorescent signals were prominently detected in WAT and mesenchymal stem cells. Additionally, we also found that choline acetyltransferase was expressed in macrophages, suggesting their involvement in acetylcholine (ACh) secretion. Corroborating this notion, we were able to quantitatively measure the ACh in subcutaneous tissues by liquid chromatography tandem mass spectrometry. Collectively, our findings suggest that endogenous ACh released from macrophages maintains the homeostasis of adipose cell growth and differentiation via mAChR-M4 in male mice. This study provides new insights into the molecular mechanisms underlying obesity and potential targets for therapeutic interventions.

Aging is an extremely complex multifactorial process characterized by functional decline over time. Obesity and metabolic disorders that accompany aging are serious global health problems that involve both physical and emotional signs and symptoms. As many factors contribute to the age-related increase in adiposity, the accumulation of more evidence is needed to better understand the biological and pathological mechanisms underlying the excessive gain of body fat. White adipose tissue (WAT) functions as an energy reservoir and as an endocrine organ. Some cytokines are overexpressed in the WAT of obese individuals and can lead to diseases related to chronic inflammation (1). It has been shown that the age-related decline in metabolism is partly due to reduced muscle mass (2). Additionally, the age-related decline in metabolic thermogenesis in brown adipose tissue (BAT) has been strongly linked to the accumulation of body fat in mid-aged individuals (3). The discovery of effective and safe pharmacological options for preventing and treating obesity is expected to provide new avenues for improving metabolic health. However, better understanding of the pathways and mechanisms that underlie weight gain with advancing age is needed.

Muscarinic acetylcholine receptors (mAChRs) are a family of G-protein-coupled receptors that are involved in muscarinic signaling. There are 5 subtypes of mAChRs (M1-M5) and they are expressed in both the central and the peripheral nervous systems. They are also expressed in non-neuronal tissues, where they mainly play pivotal roles in the digestive system (4-7). At the molecular level, the M4 subtype of mAChR (mAChR-M4) couples to G proteins of the Gi/Go family. mAChR-M4 is expressed abundantly in the striatum, a region known to be critically involved in extrapyramidal motor control, and mAChR-M4 is known to exert inhibitory control on D1 dopamine receptor–mediated locomotor stimulation (8). In non-neuronal tissues, mAChR-M4 is expressed in both keratinocytes and melanocytes, and has been shown to control murine hair follicle cycling and pigmentation (9-11). However, the role of acetylcholine (ACh) signaling via mAChR-M4 in obesity with advancing age remains obscure.

In the present study, we employed a loss of function mouse model to investigate the role of mAChR-M4 in body weight homeostasis in adipose tissues. We found a relationship between neurosecretory protein GL (NPGL) (12) and mAChR-M4 in the central nervous system. Our results revealed a novel function of mAChR-M4 in controlling the body weight and homeostasis of adipose cell growth and differentiation.

## Materials and Methods

### Animal Experiments

#### Animals

The global M1-M5–/– (M1- to M5-KO) mice and wild-type (WT) mice on the C57BL/6 background have been described previously (8, 13-16). Male mice were singly housed or housed in a group of 3 at all times in a temperature- and humidity-controlled environment (22 °C ± 2 °C, 33-35% humidity) on a 12-hour light–dark cycle. Mice were maintained on a standard chow diet (CE-2; CLEA Japan, Tokyo, Japan). For the high-fat diet (HFD)–induced obesity experiment, age-matched WT and M4-KO mice were single-housed and maintained on a HFD in which fat accounted for 60% of the calories (HFD-60; OrientalBio, Tokyo, Japan) for 12 weeks. Body weight and food intake were measured weekly for 12 weeks. The weekly food intake was determined by subtracting the mass of the food remaining from that supplied for the singly housed mice at the beginning. All animal experiments were approved by the Suntory animal ethics committee (APRV000024 and APRV000561) and were performed according to the institutional guidelines. Mice were euthanized by CO_2_ asphyxiation.

#### Generation of *Chrm4-mScarlet* knock-in and IRES-mScarlet reporter mice

To generate a *Chrm4-mScarlet* knock-in (M4-KI) mouse line, we employed the CRISPR/Cas9-mediated KI transgenesis approach using mouse extended pluripotent stem cells (EPSCs), which have been demonstrated to be advantageous in rapidly generating fully EPSC-derived chimeric mice in the F0 generation. To enable CRISPR/Cas9-mediated insertion through homology-directed repair, we generated an all-in-1 Cas9 plasmid (#87108; Addgene, Tokyo, Japan) harboring the *SpCas9* gene, a guide RNA sequence targeting the *Chrm4* locus, and a puromycin resistance cassette for drug selection. A donor template containing homology arms covering the approximately 800 bp upstream and downstream of the *Chrm4* locus was constructed by assembling a DNA fragment encoding the *mScarlet-I* reporter gene (#85044; Addgene) with DNA sequences containing the woodchuck hepatitis virus post-transcriptional regulatory element (WPRE) and SV40 polyadenylation signal using an NEBuilder kit (#E2621; New England Biolabs, Tokyo, Japan).

To obtain KI EPSC clones in which an mScarlet-I reporter cassette was inserted into the translation initiation site of the *Chrm4* locus, we cotransfected EPSCs with the all-in-1 Cas9 plasmid along with the donor template DNA using the TransIT-2020 transfection reagent (#MIR5404; Mirus Bio, Madison, USA). After puromycin selection to enrich the transfected cells, the surviving EPSCs were further cultured on mitomycin-treated feeder cells in a 10-cm dish supplemented with N2B27 medium containing 10^3^ U/mL of leukemia inhibitory factor, 3 μM CHIR99021 (4423; Tocris, Bristol, UK), 2 μM (S)-(+)-dimethindene maleate (1425; Tocris), and 2 μM minocycline hydrochloride (M9511; Sigma, Kawasaki, Japan). The formed EPSC colonies were picked and screened by reverse transcription polymerase chain reaction (RT-PCR) genotyping to select those with correctly integrated sequences in the desired genomic locus. The EPSCs harboring the mScarlet reporter were microinjected into 8-cell stage embryos (ICR; SLC, Hamamatsu, Japan). Then, the injected embryos were transferred to the oviducts of E0.5 pseudo-pregnant females. The resulting chimeric mice that were born were used for this study.

*Chrm4-IRES-mScarlet* KI mice were generated in a similar manner as the M4-KI mice using the CRISPR/Cas9-mediated KI transgenesis approach in EPSCs. The *IRES-mScarlet-pA* cassette was inserted downstream of the stop codon to maintain the endogenous expression of *Chrm4*. As expected, the homozygous *Chrm4-IRES-mScarlet* mice were healthy and fertile.

#### Blood analysis

Random blood glucose was measured with a glucometer (Glutest Neo Alpha Sensor; Sanwa Kagaku Kenkyusho, Kyoto, Japan) using blood collected from the tail vein. For serum collection, mice were euthanized by CO_2_ asphyxiation, and blood was collected from the heart. The whole blood was kept at room temperature until it clotted, then it was centrifuged at 2000*g* at room temperature for 10 minutes to separate the serum. The serum concentrations of insulin and leptin were measured with an Ultra-Sensitive Mouse Insulin ELISA kit (M1104; MORINAGA, Yokohama, Japan) (Table 1) and Mouse and Rat Leptin ELISA kit (RD291001200R; BioVendor, Brno, Czech Republic) (Table 1), respectively, according to the manufacturer's protocols. Serum samples were stored at −80 °C until assayed. For measuring the glucagon-like peptide-1 (GLP-1) levels, blood samples were collected into lithium heparin anticoagulant tubes, and plasma was prepared by centrifugation of the samples at 2000*g* for 10 minutes at 4 °C. The concentration of GLP-1 in the plasma was measured with a GLP-1 (9-36/37) assay kit (27788; IBL, Gunma, Japan) (Table 1) according to the manufacturer's protocol. Plasma samples were stored at −80 °C until assayed.

**Table 1.** List of primary and secondary antibodies used for immunofluorescence studies

Target	Antibodies	Company	Cat#	RRID#	Final concentration
CD34	Anti-CD34 Monoclonal Antibody (RAM34), FITC	Thermo Fisher Scientific	11-0341-81	AB_465021	1/100
CD105	Anti-CD105 Monoclonal Antibody (MJ7/18), Alexa Fluor 647	Biolegend	120419	AB_2728140	1/100
Perilipin-1	Anti-Perilipin-1 Polyclonal Antibody	Abcam	ab3526	AB_2167274	1/400
RFP	Anti-RFP Monoclonal Antibody (5F8)	Proteintech	5F8-150	AB_2336064	1/250
ChAT	Anti-Choline Acetyltransferase (ChAT) Polyclonal Antibody	Merck	AB144P	AB_2079751	1/500
F4/80	Anti-F4/80 Monoclonal Antibody (BM8), Alexa Fluor 488	Biolegend	123119	AB_893491	1/100
	eFluor 570 Mouse anti-Rat IgG2a	Thermo Fisher Scientific	41-4817-80	AB_2573615	1/1000
	Alexa Fluor Plus 488 Goat Anti-Rabbit IgG (H + L)	Thermo Fisher Scientific	A32731	AB_2633280	1/1000
	Alexa Fluor 568 Donkey Anti-Goat IgG (H + L)	Abcam	Ab175704	AB_2725786	1/1000
Leptin	Leptin Mouse/Rat ELISA kit	BioVendor Laboratory Medicine	RD291001200R	AB_2888686	
GLP-1	GLP-1 (9-36/37) ELISA kit	Tecan (IBL)	JP27788	AB_3065262	
Insulin	Ultra-Sensitive Mouse Insulin ELISA Kit	Morinaga Institute of Biological Science	M1104	AB_2811268	

Abbreviations: ELISA, enzyme-linked immunosorbent assay; FITC, fluorescein isothiocyanate.

#### Quantitative analysis of Neuropeptide Y

Neuropeptide Y (NPY) crude peptides were extracted from WT and M4-KO mouse brains as previously described (17). After solid-phase extraction, the NPY peptides were applied to gel filtration chromatography as previously described (17).

Quantitative data on NPY were acquired using Nexera Micros HPLC and LC-MS 8060 triple-quadrupole MS/MS instruments (Shimadzu, Kyoto, Japan) in which a Shim-pack MC C18 column (0.3 × 50 mm, 1.9 μm; Shimadzu) was used as an analytical column. The trap column was an InertSustainSwift C18 cartridge (2.1 × 10 mm, 5 μm; GL Sciences, Tokyo, Japan). The Nexera Micros HPLC system has a MicrosLC gradient solvent elution system for the analytical column and an isocratic elution HPLC system for the trap column. Solvents A (formic acid aqueous solution) and B (acetonitrile with 0.1% formic acid) were used in the MicrosLC. The flow rate was 14 μL/min, and the gradient program from solvent A to B was as follows: the solvent B content was increased from 22% B to 38% B over 21 minutes. The transition of the triple quadrupole MS/MS was *m/z* 712.80 to *m/z* 751.10 for NPY. The retention time was about 15.8 minutes for NPY. The crude extract (1 mL) derived from gel filtration chromatography was divided into 5 aliquots (200 µL each). Each aliquot was lyophilized using a freeze-dryer (FDU-2110; EYELA, Osaka, Japan). After the lyophilized samples were dissolved in 67 µL of water, an aliquot of 14 µL was injected into the microLC-MS/MS apparatus for quantitative analysis.

#### Gene expression analysis (qRT-PCR)

Brain tissue, subcutaneous tissue, liver tissue, and interscapular BAT were dissected from WT and M4-KO mice. They were immediately frozen with liquid nitrogen and stored at −80 °C. For the experiments, tissues were homogenized in Trizol reagent (Sepasol-RNA I Super G; Nacalai Tesque, Kyoto, Japan), then incubated for 5 minutes at room temperature. Chloroform (200 µL; Nacalai Tesque) was added, and the tubes were shaken vigorously, then incubated for 3 minutes at room temperature. The samples were then centrifuged at 18 000*g* for 15 minutes at 4 °C. The aqueous phase was mixed 1:1 with isopropanol (Nacalai Tesque), then incubated for 10 minutes on ice. Subsequently, the samples were centrifuged at 18 000*g* for 15 minutes at 4 °C. To remove genomic DNA, the pellets were treated with DNase I (TURBO DNase; Ambion, Austin, TX, USA) for 15 minutes at 37 °C. To stop the reaction and precipitate the RNA, 30 µL of nuclease-free water and 30 µL of LiCl precipitation solution were added. The samples were mixed thoroughly, then chilled for 30 minutes at −20 °C. The RNA solutions were centrifuged at 20 000*g* for 15 minutes at 4 °C to pellet the RNA. The RNA content was measured using NanoDrop (DS-11; Thermo Fisher, Waltham, MA, USA). Subsequently, the RNA (1 µg) was used as a template for cDNA synthesis. Reverse transcription was performed with SuperScript II and oligo-dT primers (3′ RACE System; Invitrogen, Carlsbad, CA, USA) according to the manufacturer's protocol.

Specific gene expression levels were analyzed by quantitative RT-PCR (qRT-PCR). The qRT-PCR for specific genes was performed in triplicate using SYBR Green Master Mix (Bio-Rad, Hercules, CA, USA) according to the manufacturer's protocols. qRT-PCR was carried out on a CFX96 Real-Time System (Bio-Rad) with the following conditions: polymerase activation and DNA denaturation for 30 seconds at 95 °C, followed by 45 cycles at 95 °C for 10 seconds and 55 °C for 30 seconds, then 65 °C for 5 seconds, and finally 95 °C for 50 seconds for the melt-curve analysis. The glyceraldehyde-3-phosphate dehydrogenase gene *(GAPDH)* was amplified as an internal control. All primers for qRT-PCR are shown in Table S1 (18). As the region between the middle of the second transmembrane domain and the N terminus of the third intracellular loop of the mAChR-M4–coding sequence was disrupted (8), we designed the primers to be the site of the disruption. For the relative quantification of the gene expression level, the ΔΔC_T_ method was used.

#### RNA sequencing

Total RNA was extracted from the subcutaneous adipose tissues of WT and M4-KO mice and purified and depleted genomic DNA as described above. Three individual samples were collected and used for RNA sequencing (RNA-seq). An aliquot (500 ng) of quality-confirmed RNA was used for library construction, and was sequenced by Novagene (Beijing, China) using the Illumina NovaSeq 6000 platform (Illumina, San Diego, CA, USA). The resulting fastq files were analyzed and deposited into the National Center for Biotechnology Information (NCBI) database (accession no. SRR29366322-SRR29366333). The reads in these fastq files were mapped to mouse mm10 genome using Hisat2 (v2.2.1) and the resulting gene expression levels are estimated as exported values of transcripts per million (TPM) using cufflinks (v2.2.1). The RNA-seq data were confirmed by qRT-PCR as described above.

#### Bioinformatics analysis

Differential expression and gene ontology (GO) enrichment analyses were conducted using iDEP.951 (http://bioinformatics.sdstate.edu/idep95/). Briefly, the normalized TPM expression values were imported into iDEP.951 and subjected to log transformation, then the transcripts with a low abundance (<1 TPM) were removed from the analysis. Differentially expressed genes (DEGs; genes with a 1.5-fold change and a false discovery rate adjusted *P* < .05) were then subjected to GO enrichment analysis using iDEP.951.

#### Immunofluorescence staining

For immunofluorescence staining experiments, subcutaneous adipose tissue was dissected from WT, M4-KI, and M4 reporter mice. The tissues were fixed with 4% paraformaldehyde (Nacalai Tesque) for 3 hours at 4 °C, followed by washing with phosphate-buffered saline (PBS; pH 7.4) and an overnight incubation in 15% (w/v) sucrose. Tissue samples were prepared for cryosectioning as described previously (19). The samples were incubated in a 15% sucrose to 7.5% gelatin solution for 1 hour at 37  °C. Then, the samples were placed in Tissue-Tek Cryomolds (Sakura Finetek, Torrance, CA, USA) filled with a warm sucrose–gelatin solution. The Cryomolds were incubated for 20 minutes at 4 °C to allow the sucrose–gelatin solution to solidify. The obtained blocks were trimmed and stored at 4 °C. The blocks were subsequently embedded in Tissue-Tek OCT compound using new Tissue-Tek Cryomolds and stored at −80 °C before sectioning (10 µm) on a cryostat (CryoStar NX70; Thermo Scientific, Waltham, MA, USA). Before immunofluorescence staining, sections were dried for 1 hour at 37 °C. Subsequently, they were washed twice with PBS and PBS containing 0.2% Triton X-100 (PBST; Nacalai Tesque) for 2 minutes and 5 minutes in series, respectively. The sections were then treated with 1% bovine serum albumin (Sigma-Aldrich, St. Louis, MO, USA) dissolved in PBST for 60 minutes at room temperature. The primary and secondary antibodies used for immunofluorescence (Table 1) were diluted with 1% bovine serum albumin/PBST.

For the double immunofluorescence staining of mScarlet and perilipin-1, sections were incubated overnight with a combination of rat anti-red fluorescent protein (RFP) antibody (1:250) and rabbit anti-perilipin-1 antibody (1:400) at 4 °C. After washing twice with PBS for 2 minutes, the sections were incubated for 1 hour with a combination of eFluor 570 mouse anti-rat IgG2a (1:1000) and Alexa Fluor Plus 488 goat anti-rabbit IgG (1:1000) at room temperature.

For the triple immunofluorescence staining of mScarlet, CD34, and CD105, sections were first incubated overnight with rat anti-RFP antibody (1:250) at 4 °C. After washing twice with PBS for 2 minutes, the sections were incubated for 1 hour with eFluor 570 mouse anti-rat IgG2a (1:1000) at room temperature. Next, the sections were incubated for 1 hour with a combination of rat anti-CD34 monoclonal antibody conjugated with fluorescein isothiocyanate (1:100) and rat anti-CD105 monoclonal antibody conjugated with Alexa Fluor 647 (1:100) at room temperature.

For the double immunofluorescence staining of F4/80 and choline acetyltransferase (ChAT), sections were incubated overnight with a combination of rat anti-F4/80 monoclonal antibody conjugated with Alexa Fluor 488 (1:100) and goat anti-ChAT antibody (1:500) at 4 °C. After washing twice with PBS for 2 minutes, the sections were incubated for 1 hour with Alexa Fluor 568 donkey anti-goat IgG (1:1000) at room temperature.

After the fluorescence staining, sections were incubated with Hoechst 33342 (1:500; AnaSpec, Fremont, CA, USA) for 20 minutes at room temperature. After washing twice with PBS for 2 minutes, the sections were mounted in VECTASHIELD Vibrance Antifade Mounting Medium (Vector Laboratories, Newark, CA, USA) under a cover glass (Matsunami, Osaka, Japan), and observed by confocal immunofluorescence microscopy (FV3000; Olympus, Tokyo, Japan).

To determine whether the increase in subcutaneous WAT mass and whitening of interscapular BAT were associated with lipid droplet hypertrophy, we performed immunofluorescence analysis of WAT and BAT using rabbit anti-perilipin-1 antibody (1:400). The acquisition of immunofluorescence images was performed using FV3000. The nuclear density of adipocytes in WAT and BAT was determined by quantifying the number of nuclei within perilipin-1 positive regions using ImageJ (https://imagej.net/ij/).

#### Oil Red O staining

To detect fat accumulation in the liver, hepatic tissues derived from WT and M4-KO mice were fixed with 4% paraformaldehyde (Nacalai Tesque) for 3 hours at 4 °C, followed by washing with PBS (pH 7.4). The tissues were subsequently embedded in Tissue-Tek OCT compound (Sakura Finetek) and stored at −80 °C before sectioning (10 µm) on a cryostat (CryoStar NX70). Sections were air-dried for 1 hour at 37 °C and then rinsed with 60% isopropanol for 2 minutes at room temperature. The sections were then stained with Oil Red O solution (FUJIFILM Wako Pure Chemical Corporation, Osaka, Japan) for 5 minutes at room temperature and rinsed with 60% isopropanol for 2 minutes at room temperature. Next, the sections were washed with tap water. Finally, the sections were mounted in VECTASHIELD Vibrance Antifade Mounting Medium (Vector Laboratories) under a cover glass (Matsunami) and observed by inverted microscope (Axio Imager A2; ZEISS, Munich, Germany).

#### Quantitative analysis of the ACh content

Extracts for the determination of the ACh content were prepared from WT and M4-KO subcutaneous adipose tissues. The tissues were ground to fine powder using TissueLyzer II (Qiagen, Tokyo, Japan) under liquid nitrogen. The obtained powders were dissolved in 1 mL of a 1:1 mixture of MilliQ water and acetonitrile (Nacalai Tesque), then vortexed. After centrifugation at 200*g* for 1 minute at 4 °C, the supernatants were filtered twice in a spin column filter (Millex-LH; Merck, Tokyo, Japan) at 90*g* for 1 minute at 4 °C, then the filtrates were analyzed by liquid chromatography tandem mass spectrometry (LC-MS/MS).

The filtrates (10 μL) were applied to a LC-MS/MS system (LCMS-8060; Shimadzu) equipped with an Intrada amino acid column (3.0 × 50 mm internal diameter, 3 μm; Imtakt, Kyoto, Japan) operated at 40 °C for chromatographic separation. The mobile phase consisted of solvent A (0.2% (v/v) formic acid, 100 mmol/L ammonium formate, pH 4.0), and solvent B (acetonitrile), and was delivered at a flow rate of 0.3 mL/min. The linear gradient used was as follows: 0 to 5 minutes, 90% to 60% solvent B; 5 to 12 minutes, 60% solvent B; 12 to 15 minutes, 60% to 30% solvent B; and 15 to 16 minutes, 30% to 90% solvent B. The electrospray ionization (positive ionization mode) mass spectrometer was operated in multiple reaction monitoring mode to observe the transition of *m/z* 146.10 to *m/z* 87.05 for ACh quantification at a collision energy of 15 (arbitrary unit). The retention time was 3.9 minutes for ACh.

### Statistical Analysis

Two-tailed unpaired Student's *t* tests were used to compare data between 2 groups. Data and statistical analyses were performed using Microsoft Excel (Microsoft Corporation, Redmond, WA, USA). Data are presented as the mean ± SD. In all cases, differences were considered significant when *P* < .05. All experiments were repeated at least 3 times.

## Results

### mAChR-M4 Deficiency Caused Weight Gain With Advancing Age

**Figure 1.**
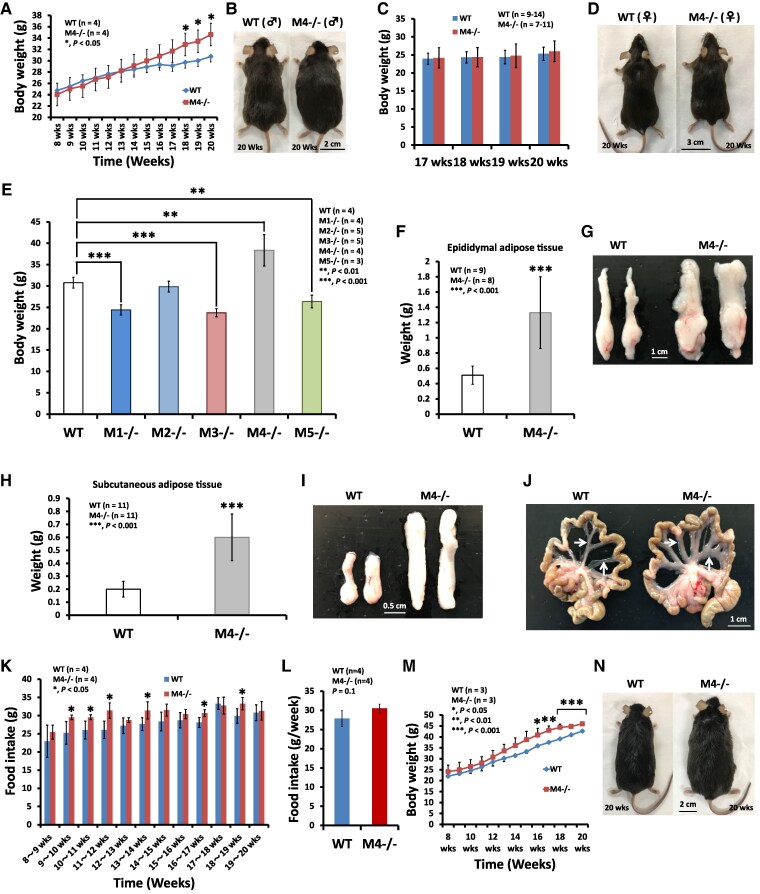
The body weight and WAT weight in aging M4–/– mice. (A) and (B) Changes in the body weight in male mice. *P < .05 for WT vs M4–/– mice. (C, D) Changes in the body weight in female mice. (E) Changes in the body weight in male mice at the age of 20 weeks. **P < .01, ***P < .001 for WT vs M1–/–, M3–/–, M4–/–, or M5–/– mice. (F, G) Epididymal adipose tissue in male M4–/– mice at the age of 20 weeks. (H, I) Subcutaneous adipose tissue in male M4–/– mice at the age of 20 weeks. (J) Mesentery adipose tissue in male M4–/– mice at the age of 20 weeks. (K) Weekly food intake of male M4–/– mice from 8 weeks to 20 weeks of age. (L) Average weekly food intake of male M4–/– mice over the experimental period. (M, N) Body weight of WT and M4–/– mice during the feeding of a high-fat diet (HFD). All data are expressed as the mean ± SD. Data were analyzed using a 2-tailed Student's *t*-test. *P < .05, **P < .01, ***P < .001.

It has been demonstrated that at around 12 weeks of age, mAChR-M4–deficient mice show an increase in basal locomotor activity and are hypersensitive to the locomotor activity stimulated through the D1 dopamine receptor (8). However, little is known about the impact of the loss of function of mAChR-M4 with age. At 18 to 19 weeks of age, the body weight of male M4-KO mice was significantly increased when compared with that of the control WT mice (Fig. 1A). Notably, 20-week-old male M4-KO mice exhibited obesity while the age-matched control mice did not (Fig. 1B). Also, the body weight of female M4-KO mice did not differ from that of the control mice at 17 to 20 weeks of age (Fig. 1C and 1D). Subsequently, we examined the body weight of the 20-week-old WT mice and mice deficient for each of the 5 subtypes of mAChR (mAChR-M1 to mAChR-M5) in only the males. The body weight of the mAChR-M4-deficient mice was significantly increased when compared with the WT mice (Fig. 1E). In contrast, body weight loss was observed in the mAChR-M3-deficient mice; this decrease in body weight was thought to be due to dramatic hypoplasia of the anterior pituitary gland associated with the greatly reduced pituitary growth hormone and prolactin levels seen in our analyses of the brain of mAChR-M3-KO mice (20). Body weight loss was also seen in the mAChR-M1–deficient and mAChR-M5–deficient mice, but the reason for the weight loss remains unclear. In M4-KO mice, the weights of the epididymal adipose tissue, subcutaneous adipose tissue, and mesentery adipose tissue were increased (Fig. 1F-1J). Next, we examined the food intake from 8 weeks to 20 weeks in the WT and M4-KO mice. Each mouse was individually housed, and the weekly food intake was measured. The food intake of the M4-KO mice was significantly higher at 9, 10, 11, 13, 16, and 18 weeks than that of the WT mice (Fig. 1K). However, almost no difference was seen in the average weekly food intake over the entire study period between the WT and M4-KO mice (Fig. 1L).

As impaired energy expenditure in WAT is closely linked to the development of obesity (21), we examined the physiological acclimation of M4-KO mice to nutrient overload. When fed the standard chow diet, the energy expenditure in WAT differed between the M4-KO and WT mice (Fig. 1A and 1B). When fed the HFD, the M4-KO mice showed greater weight gain than the WT mice even though the food intake was similar (Fig. 1M and 1N). Based on these results, we hypothesized that mAChR-M4 signaling mediated by ACh may provide essential protection against weight gain with advancing age and HFD-induced obesity as well as other associated metabolic disorders, and that even partial loss of BAT and/or beige fat regulation may have serious detrimental effects on systemic metabolism.

### Deletion of *Chrm4* Induced Lipid Droplet Hypertrophy in Subcutaneous WAT, but Not in the Liver

**Figure 2.**
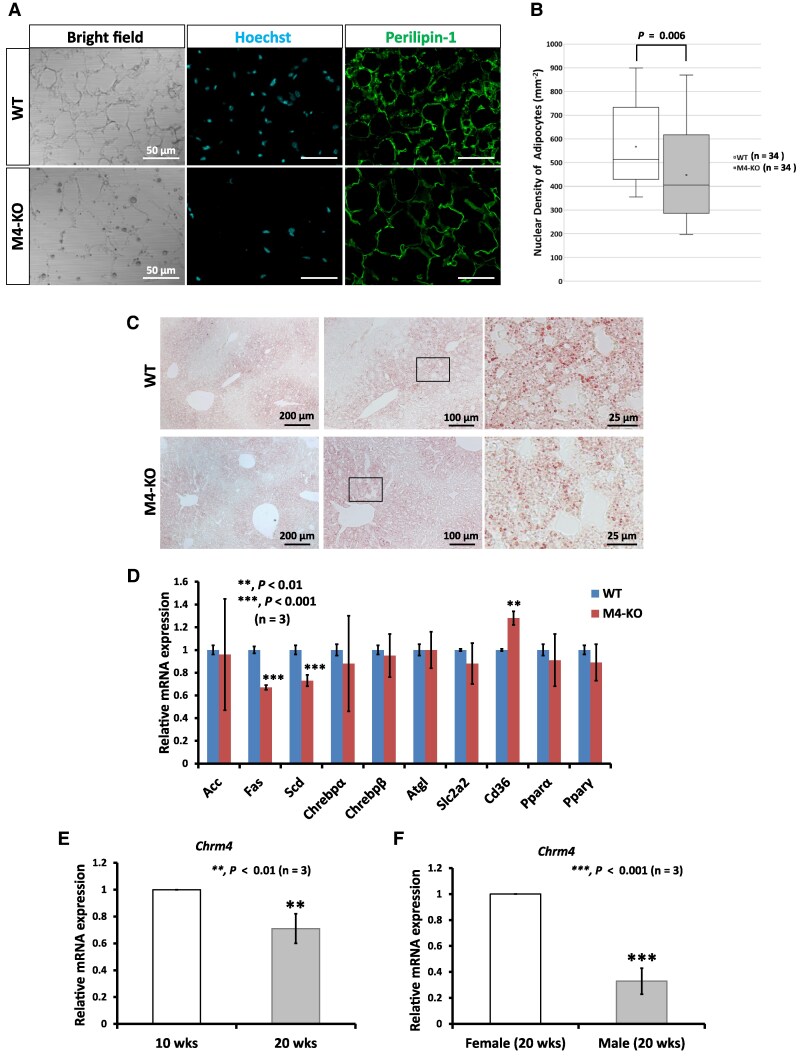
Morphological and mRNA expression analyses in the subcutaneous adipose tissue and the liver of WT and M4-KO mice. (A) Anti-perilipin-1 antibody staining of subcutaneous adipose tissue. (B) Analysis of nuclear density of the subcutaneous adipose cells in WT and M4-KO mice. (C) Oil Red O staining of the liver sections. (D) Relative quantification of the expression level of lipid metabolism-related genes in the liver of M4-KO mice. (E) Relative quantification of the expression level of *Chrm4* in the subcutaneous adipose tissues of 10-week-old and 20-week-old male WT mice. (F) Relative quantification of the expression level of *Chrm4* in the subcutaneous adipose tissues of male and female WT mice at 20 weeks of age. The results of qRT-PCR are based on 3 independent experiments and are presented as the mean ± SD. Data were analyzed using a 2-tailed Student's *t*-test. **P < .01, ***P < .001.

We next performed immunofluorescence analysis of subcutaneous WAT to determine whether the increase in WAT mass was associated with lipid droplet hypertrophy. Anti-perilipin-1 antibody staining revealed that adipocyte in subcutaneous WAT of 20-week-old male M4-KO mice was enlarged compared with that of WT male mice (Fig. 2A). Additionally, we confirmed that nuclear density of adipocytes was significantly decreased in M4-KO mice (Fig. 2B). The results revealed that the droplet in subcutaneous WAT was hypertrophic (Fig. 2A and 2B). As liver is the chief organ of lipid metabolism, we then examined the effect of *Chrm4* deletion on liver lipid accumulation. Oil Red O staining revealed that hepatic fat content in M4-KO mice was not different from that in WT mice despite adiposity in the subcutaneous WAT (Fig. 2C). Gene expression analysis was performed to determine whether deletion of *Chrm4* alters the gene expression pattern of lipid metabolism–related genes in the liver. The qRT-PCR analysis revealed that the gene expression patterns for *Fas* and *Scd* were significantly decreased, while the gene expression of *Cd36* was significantly increased (Fig. 2D). However, gene expression patterns for other genes were not significantly changed (Fig. 2D). These data suggest that aberrant lipid metabolism in liver may not occur in M4-KO mice.

We found that mAChR-M4 deficiency caused weight gain with advancing age. Accordingly, we examined to determine whether *Chrm4* expressions decreased with advancing age in WT mice. *Chrm4* expression in subcutaneous WAT of 10-week- and 20-week-old male WT mice was examined with qRT-PCR. The result showed that *Chrm4* expressions were significantly decreased accompanied by the aging process (Fig. 2E). Additionally, we examined to determine whether there was difference between the expression of *Chrm4* in the subcutaneous WAT of male and female WT mice at 20 weeks of age. The result showed that *Chrm4* expression in subcutaneous WAT of male mice was significantly decreased (Fig. 2F). These data indicate that sex-related decrease of *Chrm4* expression solely occurs in male mice.

### Serum Leptin Levels in Peripheral Metabolism Were Increased in M4-KO Mice

mAChR-M4 is expressed abundantly in the striatum and is also present at low levels in several other brain regions, such as the cerebral cortex and hypothalamus (22-25). As M4-KO mice exhibited body weight gain and WAT accumulation, we hypothesized that communication between the central nervous system, including the hypothalamus, and peripheral metabolic tissues was impaired due to the lack of mAChR-M4 in the whole body. To investigate the mediators of interorgan communication, we monitored the blood glucose, serum insulin, serum leptin, and plasma GLP-1 levels in 20-week-old mice, because prolonged increases of these metabolites are known to be associated with the development of obesity. The serum leptin levels significantly increased in the M4-KO mice when compared with the control mice (Table 2). However, there was no significant difference in the blood glucose (Fig. S1 (18)), serum insulin, and plasma GLP-1 levels between the M4-KO and WT mice (Table 2). Additionally, we quantitatively measured the neuropeptide Y (NPY) level in the brain using LC-MS/MS. NPY is involved in food intake, obesity, and metabolic diseases (26). However, no significant difference was observed in the NPY level in the brains of the M4-KO and WT mice (Table 2).

**Table 2.** Analysis of the hormone and glucose levels in 20-week-old WT and M4-KO mice

	WT	M4-KO
Glucose (mg/dL)	157.2 ± 27 (n = 10)	152.6 ± 16 (n = 7)
Insulin (ng/mL)	1.4 ± 0.8 (n = 5)	2.5 ± 1.3 (n = 6)
Leptin (pg/mL)	2861 ± 1199 (n = 4)	13656 ± 7274* (n = 3)
GLP-1 (pmol/L)	4.1 ± 1.8 (n = 3)	5.6 ± 4.8 (n = 5)
NPY (pmol/brain)	9.3 ± 3.1 (n = 6)	9.1 ± 0.8 (n = 6)

The biochemical parameters of the plasma and brain were analyzed in 20-week-old WT and M4-KO mice. Data are shown as the mean ± SD. Statistical significance was calculated using a 2-tailed Student's *t*-test (*P < .05).

Abbreviations: GLP-1, glucagon-like peptide 1; M4-KO, *Chrm4*-knockout; NPY, neuropeptide Y; WT, wild type.

**Figure 3.**
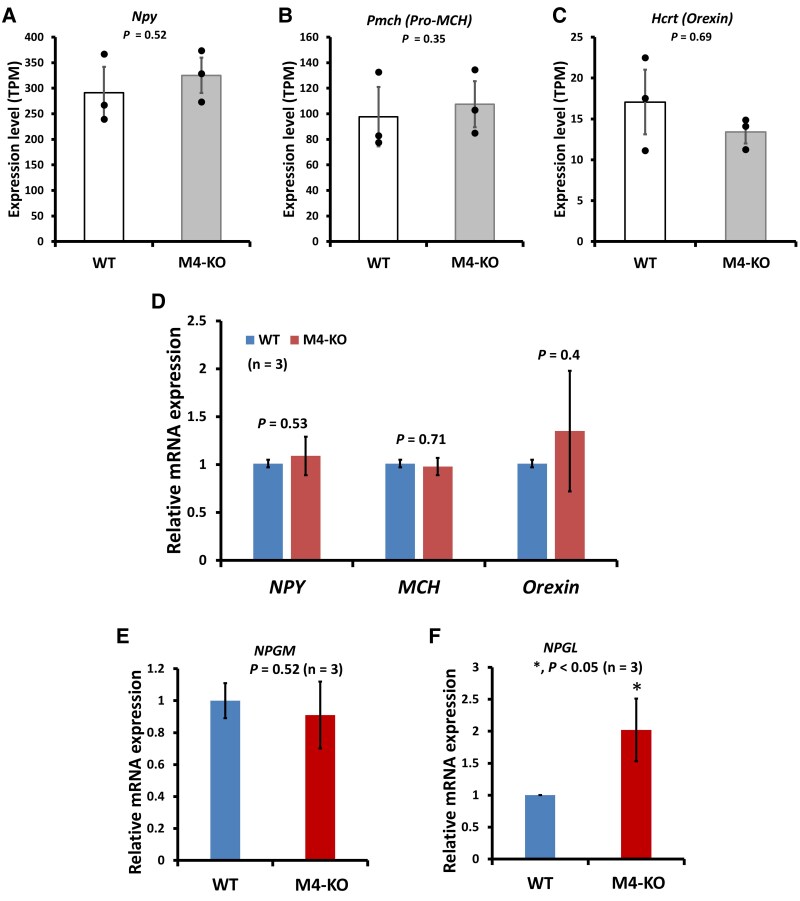
Comparison of the expression of marker genes in the brain of WT and M4-KO mice. (A-C) Differential expression patterns of *Npy*, *Pmch* (*Pro-Mch*), and *Hcrt (orexin)* in the brain of M4-KO mice. The results of RNA-Seq are based on 3 independent experiments and are presented as the mean ± SD. (D) Relative quantification of the expression level of *Npy*, *Mch*, and *Hcrt* in the brain of M4-KO mice. (E, F) Relative quantification of the expression level of *NPGM* and *NPGL* in the brain of M4-KO mice. The results of qRT-PCR are based on 3 independent experiments and are presented as the mean ± SD. Data were analyzed using a 2-tailed Student's *t*-test. *P < .05.

The brain, particularly the hypothalamus, regulates leptin signaling by sensing leptin hormone, and by integrating and coordinating neurophysiological responses to control whole-body metabolism. To investigate the underlying cause of the energy homeostasis in the M4-KO mice, gene expression in the whole brain was examined by RNA-seq and qRT-PCR. There was no difference in the mRNA expression levels for genes related to energy homeostasis, including for the genes encoding NPY, melanin-concentrating hormone (MCH), and orexin, between the M4-KO and WT mice (Fig. 3A-3D; Table S2 (18)). Interestingly, in the brain of M4-KO mice, the expression level of the gene encoding NPGL was elevated, but that of the gene encoding neurosecretory protein GM (NPGM) was not (Fig. 3E and 3F). Both neurosecretory proteins have been shown to be essential for energy metabolism (12, 27). The increased expression of *NPGL* precursor mRNA in the brain of M4-KO mice may contribute to the obese phenotype associated with elevated secretion levels of serum leptin from WAT, which increases with WAT accumulation without any increase in dietary intake (28).

### Deletion of *Chrm4* Increased Subcutaneous WAT Marker Gene Expression and Decreased BAT/Beige Marker Gene Expression

**Figure 4.**
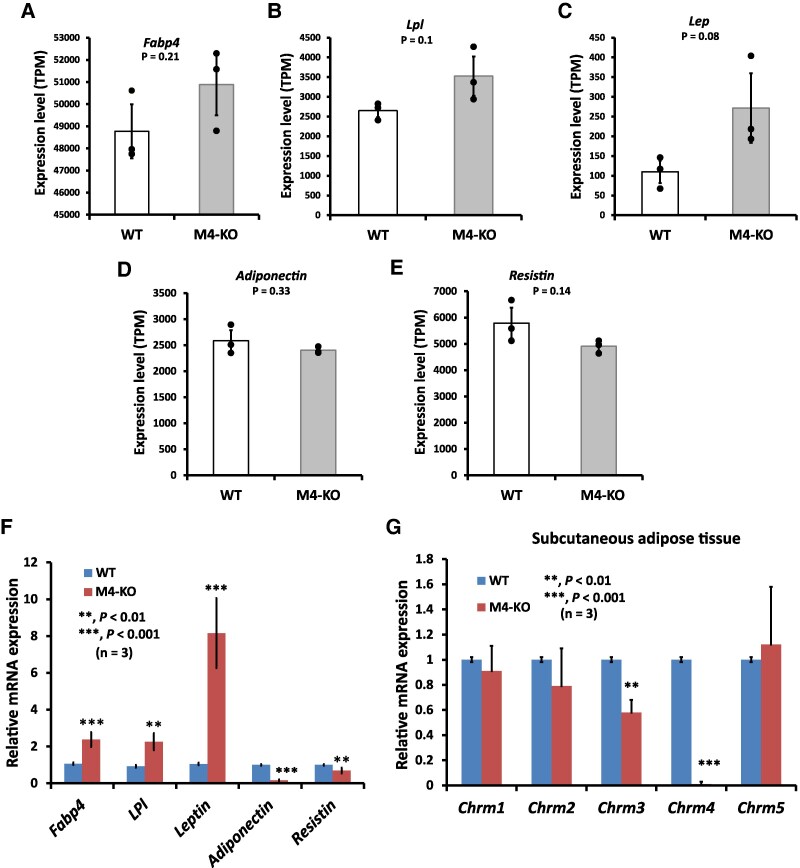
Comparison of the expression of WAT marker genes in the subcutaneous adipose tissue of WT and M4-KO mice. (A-E) Differential expression patterns of *Fabp4*, *Lpl*, *Lep*, *Adiponectin,* and *Resistin* in the subcutaneous adipose tissue of M4-KO mice. The results of RNA-Seq are based on 3 independent experiments and are presented as the mean ± SD. (F) Relative quantification of the expression level of *Fabp4*, *Lpl*, *Leptin*, *Adiponectin*, and *Resistin* in the subcutaneous adipose tissue of M4-KO mice. (G) Relative quantification of the expression level of muscarinic receptor genes in the subcutaneous adipose tissue of M4-KO mice. The results of qRT-PCR are based on 3 independent experiments and are presented as the mean ± SD. Data were analyzed using a 2-tailed Student's *t*-test. **P < .01, ***P < .001.

The body weight of 20-week-old male M4-KO mice was approximately 120% that of the control mice (Fig. 1E); this increase in body weight appeared to be associated with an increase in WAT (Fig. 1F-1J). This indicated that mAChR-M4 directly targets WAT to control the body weight. To examine the molecular mechanism, we performed RNA-seq using the subcutaneous WAT of WT and M4-KO mice. The complete list of the sequenced genes is provided in Table S3 (18). As shown in Fig. 4A-4C, the mRNA expression levels of genes that are highly expressed in WAT, such as *Fabp4*, *Lpl*, and *Lep*, tended to be increased in the subcutaneous WAT of M4-KO mice. We then carried out qRT-PCR to confirm the expression levels of the genes, and found that they were significantly enhanced in the subcutaneous WAT of M4-KO mice (Fig. 4F). In contrast, the expression levels of the genes for adiponectin and resistin, which are commonly used as markers of WAT (29, 30), tended to be lower in the subcutaneous WAT of M4-KO mice at the transcript level (Fig. 4D and 4E). Accordingly, we confirmed that the expression levels of the genes were significantly decreased in the subcutaneous adipose tissue of M4-KO mice (Fig. 4F). Individuals with obesity and type 2 diabetes have low plasma adiponectin levels (31). Resistin was first discovered as an adipose-secreted hormone (adipokine) linked to obesity and insulin resistance in mice (32). The level of WAT-derived resistin is increased in obese mice and is strongly related to insulin resistance (33). However, it is possible that the serum level of resistin does not correlate with the tissue mRNA levels (34).

Gene expression analysis was performed to determine whether deletion of *Chrm4* alters the gene expression pattern of mRNAs encoding other mAChR subtypes in subcutaneous adipose tissue. Loss of expression of *Chrm4* was confirmed in the M4-KO subcutaneous adipose tissue (Fig. 4G). The qRT-PCR analysis results revealed that in the subcutaneous adipose tissue, the gene expression patterns for *Chrm1*, *Chrm2*, and *Chrm5* were similar between the M4-KO and WT mice, while the gene expression of *Chrm3* was lower in the M4-KO mice than in the WT mice (Fig. 4G). mAChR-M3 deficiency resulted in weight loss (Fig. 1E) (7). Decreased expression of *Chrm3* may compensate for the body weight gain of M4-KO mice.

**Figure 5.**
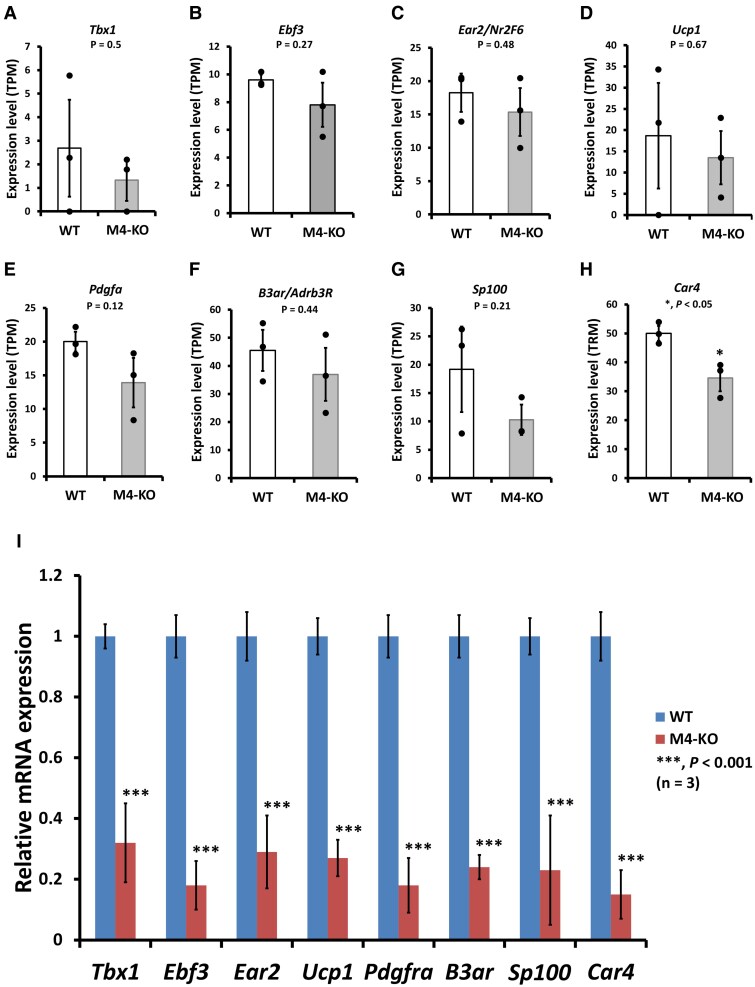
Comparison of the expression of BAT/beige adipose tissue marker genes in the subcutaneous adipose tissue of WT and M4-KO mice. (A-H) Differential expression patterns of *Tbx1*, *Ebf3*, *Ear2/Nr2F6*, *Ucp1*, *Pdgfa*, *B3ar/Adrb3R*, *Sp100*, and *Car4* in the subcutaneous adipose tissue of M4-KO mice. The results of RNA-Seq are based on 3 independent experiments and are presented as the mean ± SD. Data were analyzed using a 2-tailed Student's *t*-test. *P < .05. (I) Relative quantification of the expression level of *Tbx1*, *Ebf3*, *Ear2/Nr2F6*, *Ucp1*, *Pdgfa*, *B3ar/Adrb3R*, *Sp100*, and *Car4* in the subcutaneous adipose tissue of M4-KO mice. The results of qRT-PCR are based on 3 independent experiments and are presented as the mean ± SD. Data were analyzed using a 2-tailed Student's *t*-test. ***P < .001.

The WAT accumulation in M4-KO mice suggested a decreased thermogenic capacity, and thus, a decrease in energy expenditure in the subcutaneous WAT. Therefore, we examined the expression pattern of BAT/beige adipose tissue marker genes. Deletion of *Chrm4* tended to decrease the gene expression of the markers (Fig. 5A-5H). In particular, a statistically significant decrease was detected in the *Car4* gene expression level (Fig. 5H). We then carried out qRT-PCR analysis to confirm the expression levels of the genes. We found that as the expression levels of WAT marker genes increased (with the exception of the genes encoding adiponectin and resistin), the mRNA expression levels of the BAT/beige adipose tissue-selective thermogenic genes, such as *Tbx1*, *Ebf3*, *Ear2*  */*  *Nr2F6*, *Ucp1*, *Pdgfra*, *B3ar*/*Adrb3R*, *Sp100*, and *Car4*, progressively decreased (Fig. 5I). These data suggest the presence of decreased BAT differentiation and/or impaired WAT and beige adipocyte differentiation in the subcutaneous WAT of M4-KO mice.

### Deletion of *Chrm4* Activated Immune Cells and the Cell Cycle, and Impaired Adipogenesis

**Figure 6.**
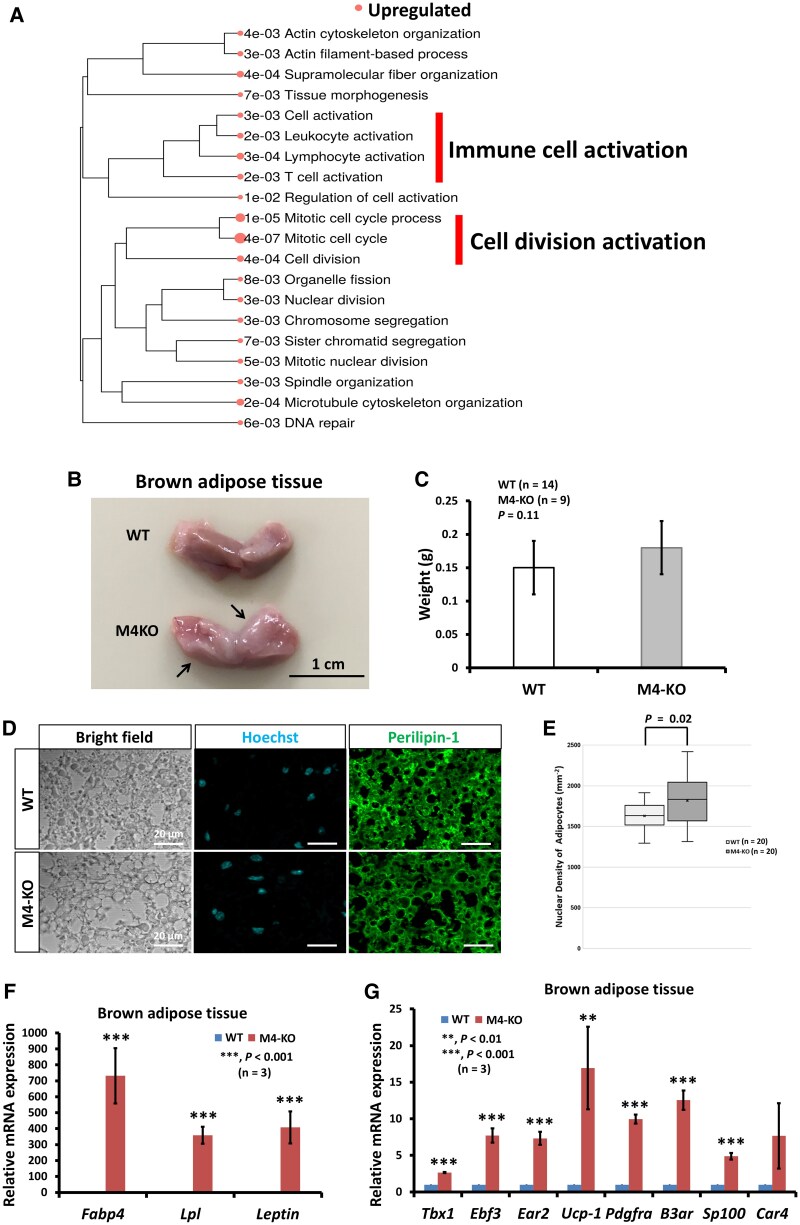
Gene set enrichment analysis of the subcutaneous adipose tissue of M4-KO mice, and comparison of the expression of WAT and BAT/beige adipose tissue marker genes in the BAT of WT and M4-KO mice. (A) GO enrichment tree shows that genes related to immune cell activation and cell division were upregulated in the subcutaneous adipose tissue of M4-KO mice. The enrichment *P*-value is indicated for each functional category. (B) and (C) The BAT in male M4-KO mice at the age of 20 weeks. (D) Anti-perilipin-1 antibody staining of brown adipose tissue. (E) Analysis of nuclear density of the brown adipose cells in WT and M4-KO mice. (F) Relative quantification of the expression level of *Fabp4*, *Lpl*, and *Leptin* in the BAT of M4-KO mice. (G) Relative quantification of the expression level of *Tbx1*, *Ebf3*, *Ear2/Nr2F6*, *Ucp1*, *Pdgfa*, *B3ar/Adrb3R*, *Sp100*, and *Car4* in the BAT of M4-KO mice. The results of qRT-PCR are based on 3 independent experiments and are presented as the mean ± SD. Data were analyzed using a 2-tailed Student's *t*-test. **P < .01, ***P < .001.

To gain a better understanding of the function of mAChR-M4 in subcutaneous WAT, upregulated and downregulated DEGs were subjected to GO enrichment analysis. The GO enrichment analysis results revealed that the upregulated DEGs were mainly enriched in immune cell activation- and mitotic cell cycle–related processes, as illustrated in the GO enrichment tree (Fig. 6A). In contrast, the downregulated DEGs showed no pattern of categorization in the GO enrichment tree.

Obesity develops through an increase in adipocyte numbers through adipogenesis and/or an increase in the size of the adipocytes. BAT plays a key role in thermogenesis, and its activation to a state of increased energy expenditure is believed to protect against the development of obesity. The 20-week-old male M4-KO mice exhibited obesity phenotypes (Fig. 1B). We examined the shape and weight of the interscapular BAT, and the expression patterns of WAT and BAT/beige adipose tissue marker genes in the M4-KO mice. The interscapular BAT of the M4-KO mice had more profound whitening in part than that of the WT mice (Fig. 6B). However, the size of the interscapular BAT was not significantly different between the M4-KO and WT mice (Fig. 6C). We next performed an immunofluorescence analysis of interscapular BAT to determine whether whitening of BAT is associated with lipid droplet hypertrophy. Anti-perilipin-1 antibody staining revealed that there was no difference between WT and M4-KO mice in interscapular BAT (Fig. 6D). Additionally, nuclear density of the adipose cells was significantly decreased compared with that of WT mice, suggesting that the droplet in interscapular BAT was not hypertrophic (Fig. 6D and 6E). We observed a marked increase in WAT marker gene expression in the M4-KO mice (Fig. 6F). The expression levels of all the BAT/beige adipose tissue marker genes (except for *Car4)* were significantly increased in the interscapular BAT of the M4-KO mice when compared with that of the WT mice (Fig. 6G). Uncoupling protein 1 regulates the thermogenic capacity of adipocytes and contributes to the regulation of energy expenditure. These results suggest that the WAT accumulation in the interscapular BAT of M4-KO mice may result from impaired lipid metabolism with no decrease in *Ucp1* expression.

### mAChR-M4 Localized to the Subcutaneous WAT and Adipose-Derived Mesenchymal Stem Cells, and *chrm4* Deletion Had no Impact on *ChAT* Expression and the ACh Content in Subcutaneous WAT

**Figure 7.**
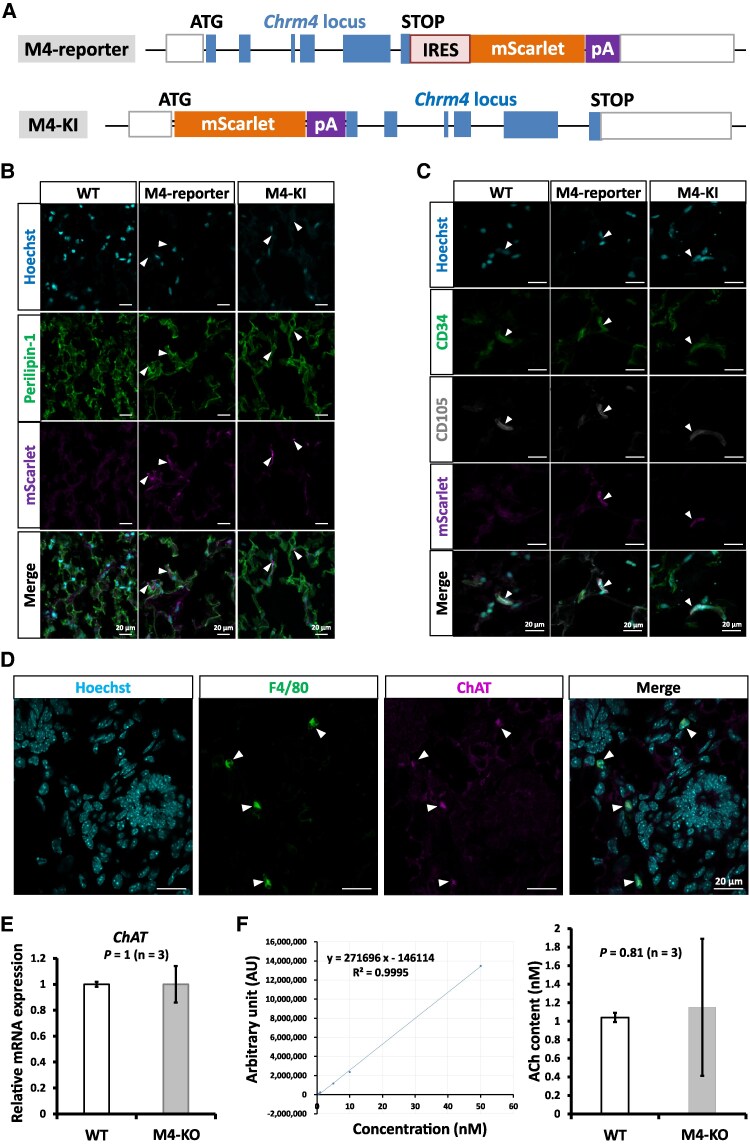
Localization of mAChR-M4 and ChAT in M4-reporter and M4-KI mice, and comparison of the expression of *ChAT* and the ACh content in the subcutaneous adipose tissue of WT and M4-KO mice. (A) Structures of transgenes integrated into the genomic DNA of M4-KI and M4-reporter mice. IRES, internal ribosome entry site. (B) Double staining using antibodies with red fluorescent protein (RFP) and perilipin-1. (C) Triple staining using antibodies with RFP, CD34, and CD105. (D) Double staining using antibodies with RFP and F4/80. Nuclei were stained using Hoechst staining. White scale bars represent 20 μm. (E) Relative quantification of the expression level of *Chat* in the subcutaneous adipose tissue of M4-KO mice. (F) The ACh content of the subcutaneous adipose tissue of M4-KO mice was measured quantitatively by LC-MS/MS analysis. The results of qRT-PCR and LC-MS/MS are based on 3 independent experiments and are presented as the mean ± SD. Data were analyzed using a 2-tailed Student's *t*-test.

To identify the cells that were expressing *Chrm4*, we employed immunofluorescence techniques to assess the cellular expression and tissue distribution of mAChR-M4. To attain a high degree of sensitivity for detection, we used the mScarlet expression system in M4-KI and *M4-IRES-mScarlet* reporter (M4-reporter) mice (Fig. 7A), which allowed detection of the endogenous expression of mAChR-M4 from the expression of mScarlet. mAChR-M4 signals were observed in a small number of perilipin-1-positive adipose cells (Fig. 7B). Additionally, in triple-staining experiments carried out to see whether mAChR-M4 localized to the adipose-derived mesenchymal stem cells (AD-MSCs), mAChR-M4 signals were also observed in a small number of CD34 and CD105-double positive cells (Fig. 7C).

We next attempted to identify the source of the ACh that acted on mAChR-M4 in subcutaneous WAT. Double-staining experiments and confocal microscopy analysis results revealed that ChAT-positive cells infiltrating the adipose parenchyma were positive for the pan-macrophage cell surface marker F4/80, indicating that the double-positive cells were resident interstitial macrophages (Fig. 7D). To further assess ChAT activity, we examined the *ChAT* expression in the subcutaneous WAT of the WT and M4-KO mice. We found that *ChAT* was expressed in the subcutaneous WAT of both the WT and M4-KO mice with no significant difference (Fig. 7E). Next, we quantified the ACh content in the subcutaneous WAT of the WT and M4-KO mice by LC-MS/MS. Results obtained from quantitative measurements using a calibration curve demonstrated that the ACh content of the subcutaneous WAT in the WT and M4-KO mice was 1.04 ± 0.05 nM and 1.15 ± 0.74 nM, respectively, and there was no significant difference between the mice (Fig. 7F). Collectively, the data showed that ACh signaling via mAChR-M4 occurs in the subcutaneous WAT of mice. In healthy mice, the signal plays homeostatic functions that have yet to be identified.

## Discussion

Mice with whole-body KO of each of the 5 mAChRs have been created, and they showed a variety of different phenotypes. It has been demonstrated that M4-KO mice (3 months of age or older) show an increase in basal locomotor activity and are hypersensitive to the stimulatory locomotor effect of D1 receptor activation (8). In peripheral tissues, M4-KO mice present a striking hair phenotype, including retarded hair follicle morphogenesis and insufficient follicular melanogenesis, when compared with age-matched WT controls (11). In the present study, using 20-week-old M4-KO mice that exhibited obesity, we found an additional function of mAChR-M4 in peripheral tissues: our results suggested that mAChR-M4 plays a role in mediating the accumulation of WAT, and thereby controls the body weight.

Obesity is a risk factor for other chronic diseases (35). Therefore, combating obesity has been a major focus of health science. Our discovery that M4-KO mice gained weight with age via the accumulation of WAT without any increase in food intake adds to the current understanding of the etiology of obesity. Sex differences are known in several aspects of obesity and the regulation of energy homeostasis in rodents and humans (36). We found that 20-week-old female M4-KO mice showed no weight gain. Further studies are needed to address the reason why the age-related weight gain did not occur in the female mice; it may be related to female-specific events, including pregnancy.

In the current study, we also administered a HFD to M4-KO mice to see whether they would show greater weight gain. As expected, compared with the control mice, the M4-KO mice gained weight relatively quickly when fed the HFD. Interestingly, experimental evidence has indicated that several factors, including the consumption of a HFD, age, and genetics, can induce the whitening of BAT and exacerbate obesity (37-39). Indeed, the 20-week-old M4-KO mice in the present study showed profound whitening, but not BAT hypertrophy. Based on the results of our study, it appears that an increase in mAChR-M4 may increase the use of fat as an energy source.

An increasing number of neuropeptides and peptide hormones have been found to be involved in energy homeostasis by regulating the dynamic equilibrium among energy intake, storage, and expenditure (40). However, we observed no difference between the brains of WT and M4-KO mice in regard to *NPY*, *pro-MCH*, and orexin gene expression. It has been reported that cDNAs encoding the small secretory protein, NPGL were present in the hypothalamus of birds and mammals (12, 41, 42). In addition, in transgenic mice generated using the C57BL/6J strain to overexpress the *NPGL* gene (*Npgl* Tg mice), feeding of a standard chow and high-calorie diets led to obesity (43). Additionally, the same research group that identified NPGL also found a novel gene encoding NPGM, and *NPGM*-expressing neurons were found to be key hypothalamic regulators of energy metabolism, in addition to NPGL (27, 41). We found that the mRNA expression level of *NPGL* was significantly enhanced in the brain of M4-KO mice, while that of *NPGM* was not. *Npgl* Tg mice showed fat accumulation without any increase in food intake, upregulation of the mRNA expression levels for lipogenic factors in adipose tissue, or changes in blood parameters (28); these findings are similar to those seen in the M4-KO mice, indicating that *Chrm4*- and *NPGL*-expressing neurons may work together to regulate energy metabolism.

Adipose tissues are classified based on their location, and physiological and functional characteristics. We demonstrated that M4-KO mice show increased weight gain with advancing age. In addition, the upregulation of marker genes specific to WAT was detected in the subcutaneous WAT of M4-KO mice. Hyperleptinemia and leptin resistance are often observed in obese mouse models (44). The manifestation of hyperleptinemia is associated with age-related obesity. In healthy conditions, increased adiposity leads to more leptin production. Increased levels of circulating leptin act on the hypothalamus and cause metabolic shifts to regulate the energy balance (45, 46). Previous studies have shown that leptin resistance is caused by various mechanisms, including endoplasmic reticulum stress and inflammation (47, 48). The 20-week-old M4-KO mice were obese and had high serum leptin levels. It is conceivable that the M4-KO mice may develop leptin resistance before the onset of obesity. Leptin exerts its effect via 2 major neuronal populations (ie, orexigenic NPY/agouti-related protein neurons and anorexigenic proopiomelanocortin [POMC] neurons) in the arcuate nucleus of the hypothalamus (49). Deletion of *Chrm4* resulted in an obese phenotype without any changes in the food intake. It has been shown that NPGL-immunoreactive fibers form close contacts with POMC neurons in the lateral part of the arcuate nucleus, suggesting that NPGL may stimulate feeding behavior through the inhibition of anorexigenic POMC neurons (12). In addition, *Npgl* overexpression in the ICR mouse strain causes obesity with no increase in food intake (28), similar to that caused by the deletion of *Chrm4*. Therefore, the coordination between *Chrm4*- and *NPGL*-expressing neurons may mediate food intake and energy homeostasis via POMC neurons. Nevertheless, it is unclear whether the M4-KO mice developed leptin resistance in the present study, and further examinations are necessary.

BAT and beige adipose tissue are the opposite of WAT in that they promote energy expenditure and counteract the complications linked to obesity. Our RNA-seq and qRT-PCR analysis results showed that the expression levels of all marker genes for BAT/beige adipose tissue were dramatically decreased, suggesting decreased energy expenditure. It is well-known that both BAT and beige adipose tissue are subject to a whitening effect that is common in obesity; in the whitening effect, the tissues acquire unilocular fat cells that gradually lose all the brown characteristics and gain WAT characteristics (50). Recent research has demonstrated that BAT/beige adipose tissue whitening is a sophisticated metabolic complication of obesity that is linked to multiple factors, such as diet, age, genetics, thermoneutrality, and chemical exposure (51). Our data provide evidence that BAT possesses a cell-intrinsic capacity to acquire a white-like state with advancing age. The increased expression of *Ucp1* may be a compensatory mechanism against certain stresses. Interestingly, the upregulation of autophagy in the BAT of mice is consistent with the age-dependent decline of BAT activity and reduced metabolic rate (52). Future analyses of autophagy during this transition are expected to uncover the fundamental mechanisms by which mAChR-M4 controls BAT maintenance.

In rodents and humans, the mAChRs and nicotinic ACh receptors expressed in WAT are modulated by a variety of pathophysiological conditions, including the cold and obesity (53-57). In the present study, we showed that mAChR-M4 is expressed in both adipocytes and AD-MSCs in the subcutaneous WAT. AD-MSCs give rise to white, brown, or beige adipose cells. The proper adipose tissue mass and function are essential for maintaining metabolic health. As WAT lacks parasympathetic cholinergic innervation (58), the ACh acting on adipocytes via ACh receptors cannot be of neuronal origin. It has previously been reported that there are ACh-producing immune cells in WAT (57). Recently, Severi and coworkers reported that ACh is synthesized and secreted by macrophages in healthy mouse epididymal WAT (59). Our data also support the notion that macrophages synthesize and secrete ACh. The secreted ACh can subsequently diffuse through the extracellular space and affect the metabolism of adjacent adipocytes and AD-MSCs via mAChR-M4 signaling. Although we have not yet investigated the possible effects of ACh on adipocyte precursors, rodent AD-MSCs express mAChRs, and their activation affects differentiation (60, 61). It is possible that signaling through mAChR-M4 might inhibit white adipose cell differentiation.

In conclusion, we demonstrated that the deletion of *Chrm4* throughout the whole body caused obesity with advancing age without any defects in energy homeostasis or hyperphagia in mice. We also presented evidence that ACh signaling via mAChR-M4 may have beneficial effects on white adipocytes. As ACh is a potent pleiotropic molecule, different kinds of ACh receptors are likely found in all cell types. Better understanding of the molecular mechanisms of the regulation of obesity by mAChR-M4 in WAT is expected to provide novel approaches for conquering obesity with advancing age. However, more work is needed to uncover the mechanism by which appetite- and energy homeostasis–related genes, including *NPGL*, in the hypothalamus of M4-KO mice govern the risk of obesity with advancing age. Targeting a specific population of neurons by using NPGL-Cre mice to generate M4-KO mice would help clarify which neurons contribute to aberrant obesity due to dysregulated gene expression caused by the deletion of *Chrm4*.

## Data Availability

All data generated and analyzed in this study are included in this article or in the data repositories; Zendo repository for Fig. S1 and Table S1-3 (18).

## Abbreviations

ACh: acetylcholine
AD-MSC: adipose-derived mesenchymal stem cell
BAT: brown adipose tissue
ChAT: choline acetyltransferase
Chrm4: gene encoding muscarinic acetylcholine receptor M4
DEG: differentially expressed gene
ESPC: extended pluripotent stem cell
GAPDH: glyceraldehyde-3-phosphate dehydrogenase
GLP-1: glucagon-like peptide-1
HFD: high-fat diet
GO: gene ontology
KO: knockout
KI: knock-in
LC-MS/MS: liquid chromatography tandem mass spectrometry
mAChR: muscarinic acetylcholine receptor
mAChR-M4: muscarinic acetylcholine receptor M4
MCH: melanin-concentrating hormone
NPGL: neurosecretory protein GL
NPGM: neurosecretory protein GM
NPY: neuropeptide Y
PBS: phosphate-buffered saline
PBST: PBS containing 0.2% Triton X-100
POMC: proopiomelanocortin
qRT-PCR: quantitative reverse transcription polymerase chain reaction
RFP: red fluorescent protein
TPM: transcripts per million
WAT: white adipose tissue
WT: wild type
